# Research on the Influence Mechanism of Dual Leadership on the Constructive Deviant Behavior of the New Generation of Employees—The Chain Mediating Effect of Promoting Regulatory Focus and Role Width Self-Efficacy

**DOI:** 10.3389/fpsyg.2021.775580

**Published:** 2021-11-29

**Authors:** Yan Zhou, Huichi Qian

**Affiliations:** Beijing Technology and Business University, Beijing, China

**Keywords:** dual leadership, promoting regulatory focus, role width self-efficacy, constructive deviance, new generation of employees

## Abstract

Under the background of innovation to win, employees’ constructive deviant behavior has become an important way for organizations to break through the bottleneck of change and realize transformation and upgrading. At present, it has become the focus of academic and practical circles. Based on cognitive evaluation theory and social cognitive theory, this study explores the impact mechanism of dual leadership on employees’ constructive deviant behavior. Using hierarchical regression analysis and bootstrap method, this article empirically tests the questionnaire data of 333 new generation employees. It is found that dual leadership has a significant positive impact on constructive deviant behavior; role width, self-efficacy, and promoting regulatory focus play a complete intermediary role between dual leadership and constructive deviant behavior. Promoting regulatory focus and role width self-efficacy play a chain intermediary role in the action path of dual leadership on constructive deviant behavior. The research results enrich the theoretical framework of employees’ constructive deviant behavior from the perspective of leadership style, and provide a practical reference for leaders to effectively guide employees to make constructive deviant behavior.

## Introduction

In the global economic era, the business environment and technological development have undergone drastic changes. Enterprises are facing the dual pressure of reform, innovation and fierce competition. Under the complex and changeable background, enterprises need to build core competitive advantages to meet the requirements of internal and external environmental changes (Shanker et al., 2017). As the first resource of enterprise reform, employees are the innovative hope of enterprise transformation and upgrading. If employees expect to create subversive results, they need to break through their inherent concepts, take positive organizational behavior, violate the existing fixed rules of the organization, take increasing the well-being of organization members as their own responsibility, and carry out positive organizational behavior under the control of strong constructive motivation. And take the initiative to bear the risks brought by corresponding behaviors (Frazier et al., 2017). The contradiction between employees’ positive behavior motivation and non-standard initiative behavior has aroused scholars’ focus on employees’ constructive deviant behavior (Mertens et al., 2016). Constructive deviant behavior refers to the positive organizational behavior of employees committed to taking organizational well-being as their own responsibility, actively solving problems, adopting out of role behavior that deviates from the rules, and realizing the purpose of organizational change and innovation (Dahling and Gutworth, 2017). Behind the motivation of stimulating constructive behavior, employees also bear the risk and pressure of challenging authority beyond their level. The study found that this constructive adventurous spirit is closely related to the differences of individual characteristics of different types of employees (Frazier et al., 2017). Therefore, this study takes the new generation of employees as the research object. Compared with other employees, the new generation of employees have stronger self-awareness and subjective initiative, are also more creative and innovative, and are committed to pursuing the sense of achievement of self-value realization. Therefore, the new generation of employees are more inclined to carry out constructive deviant behavior.

In the increasingly fierce environment of economic globalization and competitive globalization, scientific leadership is the key to guide enterprises to effectively build core competitiveness, stimulate employees’ positive organizational behavior, and then realize the transformation and upgrading of enterprises. On the one hand, with the development of comprehensively deepening innovation strategy, organizations begin to pay attention to risky transformational innovation. On the other hand, enterprises pay attention to the implementation of sustainable development strategy and emphasize steady and gradual transformation. Therefore, the dual leadership mode meeting the requirements of organizational dual development came into being. Different from a single leadership style, dual leadership requires to integrate the diversified tasks of the organization according to the classification of paradoxical thinking, effectively balance and coordinate the conflicts between tasks by exploring the internal relations between independent things, give full play to the synergistic effect of organizational duality, and realize the harmonious and stable development of the organization (Zhang et al., 2015). Because employees’ positive organizational behavior is mainly dominated by psychological cognition and behavioral motivation, this study selects the open-closed leadership style from the cognitive perspective to enrich the research results in related fields. The open leadership behavior from the cognitive perspective encourages employees to explore and innovate actively. The closed leadership behavior emphasizes that employees abide by the rules and complete their own work. The open and closed leadership behavior from the cognitive perspective can guide leaders and employees to switch to a more flexible work mode. The research on dual leadership style is deepened from the macro perspective to the micro perspective. The mechanism of influencing employees’ behavior motivation is also more in-depth and complex. According to cognitive evaluation theory, the controlling or informational characteristics of external conditions can weaken or enhance individual internal motivation, and then affect employee behavior. The open and closed dual leadership model can not only meet the individual needs of employees to actively explore, but also supervise employees to abide by the rules and regulations of the organization. In this case, employees will have a positive evaluation and response to the perception of the flexible mechanism of dual leadership, so as to stimulate employees’ positive and constructive behavior motivation. The existing research results on the impact mechanism of dual leadership are very scarce, and the research on how to promote the constructive deviant behavior of the new generation of employees is closer to the blank. Therefore, the research on the impact of dual leadership on employees’ positive organizational behavior has become a research hotspot of human resources and organizational behavior (Hunter et al., 2017).

From the perspective of constructive deviant behavior, the contradiction between the constructive positive purpose of the new generation of employees and the deviant behavior against the rules is the focus of scholars. On the one hand, constructive behavior promotes organizational change and innovation (Mertens et al., 2016), on the other hand, deviant behavior brings pressure and risks to their own career development. Therefore, whether employees will take constructive deviant behavior largely depends on the influence of individual preferences. As an adaptive mechanism under the requirements of individual working environment, regulation focus can better restore the individual’s cognitive regulation mechanism at work, and different psychological needs can stimulate employees’ different behavioral motives. Different incentives will strengthen employees’ different adjustment focus tendencies. Open leadership encourages employees to challenge risks, explore independently, provide employees with independent thinking space, promote employees to actively carry out self-assessment and adjustment, and enhance the internal motivation of employees’ proactive behavior (Tung, 2016). Closed leadership emphasizes formulating rules and regulations, supervising employees’ work progress, reducing accidents and risks caused by out of control plans (Hannes et al., 2015), meeting employees’ pursuit of creativity and promoting the improvement of employees’ innovation efficiency (Zacher and Wilden, 2014). The complementary model of dual leadership can stimulate the best effect of employees’ positive psychological motivation (Gebert et al., 2010). Employees with high promotion regulatory focus dare to take risks to achieve their “ideal” self, tend to set challenging goals, believe that they have the ability to achieve challenging goals (Tumasjan and Braun, 2012), and have a high controllable evaluation of creative behavior. Focus on maximizing “gain” (Wallace et al., 2016). Therefore, employees with higher focus on promoting regulation have higher ability and motivation, dare to take risks, break outdated organizational rules, and actively implement constructive deviant behaviors. The synergy advantage of leadership is conducive to activate the individual’s promoting regulatory focus characteristics, and effectively strengthen employees’ constructive behavior tendency with the support of strong behavioral motivation.

From the perspective of dual leadership, dual leadership not only encourages employees to actively explore, but also pays attention to guiding employees to use existing knowledge to complete established work tasks, and can flexibly switch between the two activities (Rosing et al., 2011). Employees who complete different types of work tasks can gain more confidence and sense of achievement, and stimulate employees’ self-efficacy perception. Self-efficacy is an important concept of social cognitive theory. It is an individual’s self-estimation of the ability required to complete an activity. From the perspective of dual leadership, seemingly conflicting goals and comprehensive and unified strategies are matched harmoniously. Dual leadership’s demand for employees’ work skills is not limited to existing work tasks, but also for transferable. The constantly updated knowledge and skills put forward new requirements. According to the social cognitive theory, in this case, the complex cognitive ability and behavioral motivation of employees’ perception and pursuit of elements contrary to each other are stimulated. In order to adapt to this diversity and uncertainty, employees are committed to breaking the existing role boundaries to adapt to the changing development needs under the stimulation of self-efficacy. Then it stimulates the role width self-efficacy. The role width self-efficacy is employees’ perception of the ability to complete more extensive and active roles beyond the established responsibilities, which puts more emphasis on employees’ broader role competence (Parker and Sharon, 1998). The existing research on constructive deviant behavior mainly includes cross-level comprehensive research on constructive deviant behavior, but there is less research on the interaction mechanism between its influencing factors. According to the theory of cognitive assessment, cognition, environment and behavior interact with each other. Positive psychological motivation is an important reason for the initiative behavior of employees. Among them, the promotion focus of adjustment focus promotes the production of employees’ initiative personality (Pham and Avnet, 2004), while active personality is one of the important sources of role width self-efficacy (Parker and Sharon, 1998). Three therefore, by activating the facilitative regulatory focus trait, employees may produce role width self-efficacy and positive organizational behavior under the influence of constructive motivation. Therefore, in order to explore the internal mechanism of dual leadership on constructive deviant behavior, based on cognitive evaluation theory, this article focuses on the chain mediating role of regulatory focus and role width self-efficacy.

The existing studies lack the mechanism of dual leadership affecting constructive deviance, and most other relevant research results are limited to theoretical exploration and lack of empirical test. This study is committed to revealing the “black box” of the mechanism of dual leadership affecting individual constructive deviance from the perspective of cognitive evaluation theory and social cognitive theory. With the adjustment focus and role width self-efficacy as the intermediary, explore the motivation mechanism to induce individual constructive deviant behavior, enrich the theoretical results of leadership employee relationship research and expand relevant research boundaries, so as to provide valuable practical guidance for the new generation of employees in enterprise management.

To sum up, the research goal of this article is to explore the mechanism model of dual leadership model affecting employees’ positive organizational behavior, comprehensively adopt the cognitive emotional dual path research paradigm, and explore the specific impact of employees’ psychological incentive tendency on the relationship between leadership and employees’ behavior. Specifically explain how the scientific and flexible open leadership closed dual leadership model can finally achieve the dynamic management goal of promoting employees’ positive organizational behavior by stimulating employees’ positive emotion and psychological cognition, and guide employees’ construction deviant behavior to embark on a formal and benign development path. On the one hand, it enriches the theoretical achievements in the field of human resource management and organizational behavior. On the other hand, it helps enterprises adapt to the complex and changing global economic environment and meet the win-win development needs of enterprises and employees.

## Theoretical Research and Basic Assumptions

### Dual Leadership

The paradoxical demand of the competitive nature of exploration and development leads to the restriction of key resources and tight availability of the organization. The dual leadership develops according to the paradoxical cognition on the basis of solving the problem of organizational duality. Drucker once proposed that the superstition about the authority of one person’s leadership is the main reason for the trouble of work position. Dual leadership responded to scholars’ calls for innovation paradox and leadership matrix structure (Drescher et al., 2014). The research on dual leadership is still in its infancy. The main point is to choose the contingency leadership model to solve the objective contradictions existing in the organization according to the Oriental yin-yang theory and the Western dual theory, combined with paradoxical thinking, so as to meet the organization’s multi-objective needs and complex management situations (Gibson and Birkinshaw, 2004). On the one hand, it is known that the research on dual leadership mainly includes open leadership and closed leadership from the cognitive perspective (Hannes et al., 2015), specifically using paradoxical thinking to deal with the contradictions between exploration activities and utilization activities in the organization, so as to obtain short-term benefits and long-term competitive advantage; and authorized leadership and command leadership from the perspective of power (Martin et al., 2013), which means that employees can not only plan independently, but also formulate a work framework for employees, so as to balance the contradictions caused by improper decentralization and centralization of power. Finally, transformational leadership and transactional leadership from the perspective of customary rules not only standardize employees’ work according to customary rules, but also break through the shackles of norms to stimulate employees’ subjective initiative. As the psychological cognitive mechanism is an important mechanism for leaders to affect employees’ behavior, the research on the influence of open leadership closed leadership on employees’ deviant behavior from the cognitive perspective is a hot spot in the field of organizational behavior. At the same time, with the development of positive psychology, the positive psychological motivation behind positive organizational behavior has become one of the most worthy topics to be discussed. Therefore, I support the research on the positive impact of dual leadership model on enterprises and employees from the perspective of cognition.

On the other hand, the existing research on dual leadership mainly focuses on combing the outcome variables and impact mechanisms of dual leadership. The existing impact mechanisms of dual leadership include internal motivation mechanisms such as self-efficacy, psychological empowerment (Lee and Lee, 2012), self-regulation mechanism (Tung, 2016). The research on the path of psychological motivation of organizational behavior combined with cognition emotion is the key to create innovation. The rise of positive psychology, such as psychological capital, provides a solid theoretical basis for relevant research. The internal incentive mechanism that causes positive psychological motivation provides a clear direction and interesting value point for my research. I agree with their ideas theoretically, (Lee and Lee, 2012) and others’ academic research is worthy of continuation and in-depth discussion. Dual leadership (Rosing et al., 2011) is the integration mechanism of positive organizational behavior, organizational learning (Luo et al., 2016), team dual culture. As well as other factors, dual leadership is also conducive to promoting knowledge exchange and thinking collision and realizing effective communication within the organization (Mom et al., 2009). From the perspective of psychological cognition, Bosch’s dual leadership model of open closed leadership advocates the coexistence of system and freedom, and the interaction with other environmental factors of the organization will have an important impact on employees’ behavior. The research of Luo et al. (2016) shows that dual leadership is conducive to stimulating the enthusiasm of learning and cultural exchange of the organization, and (Mom et al., 2009) proposed that the improvement of communication is also one of the manifestations of the improvement of the overall cultural mobility of the organization. I support the theoretical research with rigorous analysis and clear logic, and I am deeply inspired by it and committed to enriching the relevant theoretical framework.

### Constructive Deviance

Constructive deviant behaviors are deviant behaviors conducted by employees under the control of positive motivation for the noble intention of taking the organization as their own responsibility in order to increase the well-being of the organization and employees (Frazier et al., 2017). Employees’ compliance with the organization’s norms is different from destructive deviant behavior, which adds constructive positive psychological motivation. The existing research on constructive deviant behavior mainly focuses on the antecedent variables of influence. At the individual level, extroversion personality and initiative personality in the big five personality can effectively predict constructive deviant behavior (Crant and Wang, 2011), sense of responsibility can stimulate employees’ enthusiasm for constructive deviant behavior (Vadera et al., 2013), organizational identity, and psychological empowerment (Dahling and Gutworth, 2017). The research on relevant personality traits at the individual level is consistent with the behavioral motivation logic of CCB’s deviant behavior. Crant and Wang (2011) and other studies explain the personality reasons why the new generation of employees are more prone to constructive deviant behavior than older employees. Extroversion and initiative personality traits are important factors to stimulate positive psychological motivation, and sense of responsibility (Vadera et al., 2013). The research results of and psychological empowerment (Dahling and Gutworth, 2017) have further confirmed the close relationship between employees’ constructive deviant behavior and positive psychological factors. I highly recognize and agree with them in terms of theoretical basis and practical application, which provides a well-based research paradigm for follow-up research. And transformational emotional commitment are individual trait variables with positive impact. The impact results at the team level include team attachment, team satisfaction and team identity, which further strengthen the emotional relationship between employees and teams (Morrison, 2006). At the same time, helping culture helps to stimulate employees’ motivation for positive organizational behavior and has a significant impact on team members’ deviant behavior in construction (Mueller and Kamdar, 2011). In addition, the interaction between team characteristic variables and constructive deviant behavior is also the focus of the research. The team level is the research and development of deviant behavior at the individual level, which proves that positive interpersonal connection, i.e., positive psychological and emotional connection, is the key to stimulate employees’ constructive deviant behavior. Whether it is an individual or a team, a comfortable humanistic environment is a necessary condition for psychological comfort. The harmonious support of the whole team plays a vital role in the positive psychological motivation of employees. The subjective initiative of employees to actively carry out positive organizational behavior is stimulated, which further starts my cognition of constructive deviant behavior and positive psychological motivation. From the perspective of team interaction, we can more intuitively and reasonably explain employees’ behavioral motivation. Therefore, I support the relevant research conclusions. From the organizational level, the innovation atmosphere helps to promote employees’ constructive deviant behavior, the sense of organizational support encourages employees to actively engage in active behavior (Madjar et al., 2011), and the relationship between superiors and subordinates, authentic leadership can effectively predict employees’ construction deviant behavior. From the research results, constructive deviant behavior has a certain double-edged sword effect, so the situation of scientific guidance and support is the key to motivate employees to carry out constructive deviant behavior. To sum up, the organizational level further extends the good environmental atmosphere on the basis of the team level, which affects the practical application of employees’ positive and constructive deviant behavior, Madjar et al. (2011). The research conclusion of explains the reasons for the different performance of employee behavior in different organizations, which is consistent with the research results at the individual level and team level. It provides a very important reference for me to study the impact of organizational interaction factors on employee behavior at the enterprise level, and enriches the research of positive organizational behavior on the basis of positive psychology theory. On the basis of cognitive and behavioral motivation and environmental interaction, I agree with Madjar et al. (2011).

### Dual Leadership and Constructive Deviant Behavior

Dual leadership behavior is a dynamic model that adjusts and changes according to the situation (House, 1996). Based on paradoxical thinking, it flexibly adjusts the balance of mutually exclusive organizational activities, adapts to the unstable environment of complex changes, and meets the differentiated needs of organizational development (Rosing et al., 2011). Dual leadership emphasizes paradoxical thinking and inclusive psychology to coordinate contradictions, and is committed to achieving contradiction balance to enhance leadership. It is a combination of dual theory and leadership theory that seems to be contradictory but actually complement each other. Among them, the differences between open and closed leadership from the cognitive perspective complement each other, the observation of the overall management environment and the guidance of work behavior are more detailed, and open leadership Stimulate employees’ inspiration and thinking, encourage employees to explore bravely without fear of failure, and promote the absorption and transformation of new knowledge in the organization. Closed leadership focuses on work objectives and rules and regulations, avoid confusion and accidents in work plans, and minimize organizational risks (Gebert et al., 2010). The dual leadership style of opening and closing can effectively bring into play the synergy effect of leadership, meet the paradoxical needs of enterprise development, and stimulate employees’ positive organizational behavior. Constructive deviant behavior is an active behavior outside the role of employees who actively violate organizational rules, devote themselves to solving problems and improving the situation, and voluntarily make constructive contributions to the organization. The dual nature of motivated altruism and way violation is an important driving force for organizations to achieve breakthrough change. On the one hand, open leadership allows employees to put forward and experiment with new ideas of risk, and encourages employees to break the inherent system and achieve self-breakthrough (Rosing et al., 2011), employees feel the spiritual encouragement and resource support of open leadership. According to the cognitive evaluation theory, the controlling or informational characteristics of external conditions can weaken or enhance individual internal motivation, and then affect employee behavior. Open leadership gives employees the freedom of autonomy and innovation for active change, and can enhance employees’ internal motivation for active organizational behavior opportunity. Employees have a sense of belonging and dependence on the organization, which stimulates the sense of “ownership” of the organization as their own responsibility, adheres to a consistent position with the interests of the organization, and is committed to abiding by broader norms rather than general organizational rules, so as to dare to engage in constructive deviant behavior. On the other hand, closed leaders act according to plans and rules (Evans, 1996), pay attention to the final completion effect of tasks and objectives, pay attention to the implementation effectiveness of innovative activities, tend to improve the time efficiency of employee activity plan, and emphasize effective control and timely correction of employee behavior to ensure the predictability and utilization value of employee innovative behavior results. According to cognitive evaluation theory, employees perceive the relationship between closed leadership and rules and results. It alleviates the confusion caused by employees’ excessive freedom under open leadership, and employees are more cautious in predicting and controlling the results of constructive behavior plans. Closed leadership pays attention to the characteristics of efficiency and results, which soothes employees’ anxiety about the pressure and risk of deviant behavior, employees pay more attention to the potential benefits brought by the implementation of constructive behavior to the organization, so as to stimulate employees’ motivation for constructive deviant behavior. Open closed leadership not only gives employees a certain freedom of initiative organizational behavior, but also sets reasonable and effective control norms for employees. At the same time, it provides employees with a sense of support and security, which is conducive to employees’ initiation and feedback to the organization, the willingness to take the initiative to undertake tasks beyond the role of work responsibilities, and finally lead to constructive deviant behavior. Based on this, the following assumptions are put forward:

H1: Dual leadership has a positive impact on employees’ constructive deviant behavior.

### Mediating Role of Promoting Regulatory Focus

Although employees’ constructive deviant behavior is based on the interests of the organization, due to the constraints of resources and environment, they can only take deviant ways to realize the idea of constructive behavior and bear a certain degree of career development risk (Galperin and Bella, 2012). There have been studies on trait antecedents of constructive deviant behavior, including personality type, self-worth, network ability and other individual trait factors, it ignores the impact of individual self-regulation process on employees’ constructive deviant behavior at work, and fails to truly and comprehensively reflect the occurrence mechanism of leadership style affecting employees’ behavior at work. Employees’ implementation of constructive deviant behavior needs their own strong internal psychological motivation, and employees need to be consistent with positive self-concept. In this case, leaders’ recognition and support it plays an important role in stimulating employees’ positive psychological cognition (Mertens et al., 2016). At the same time, with the development of the era of knowledge economy, leaders no longer play the role of strict controller, but change into the role of flexible trainer, giving employees the necessary resources and space support for work and innovation. In this environment, the reasons for employees’ different behavior orientation stem from the differences of individual characteristics, and the individual adjustment focus is regarded as the individual’s self-adaptive in the environment. Mechanism is of great significance to clarify the internal mechanism of leadership affecting employees. Because the regulatory focus of trait is the stable mode and self-regulation tendency of acting and looking at problems gradually formed by individuals in the process of growth. From the perspective of individual idiosyncrasy, individuals with promotional regulatory focus desire or ensure that income is dominant, tend to accept challenges and pursue ideals, are not afraid of unknown risks, pursue active change, care about hope, desire and desire, and focus on “acquisition.” It is considered that it is more attractive to obtain the reward or achievement to achieve the goal than to avoid the cost of possible loss (Wallace et al., 2016). Therefore, when the adjustment focus tendency of leadership behavior and employees’ characteristics is consistent, employees’ cognition and motivation are strengthened to the greatest extent. Open leadership encourages employees to innovate actively, promotes employees’ positive self-assessment and adjustment through open leadership behavior, and enhances employees’ internal motivation for innovation (Tung, 2016), it gives employees space and freedom to carry out creative activities, and holds an inclusive attitude toward employees’ adventures and novel proposals. Closed leaders pay attention to plans and objectives, formulate standards and monitor the completion of tasks, care about the stability of employees’ planned activities, provide controllable guarantee for employees to carry out creative activities, and ensure the smooth development of creative free work and meet their needs. It promotes employees’ pursuit of autonomy and positive results, and then promotes the improvement of employees’ creativity and innovation performance (Hannes et al., 2015). Dual leadership urges employees to actively search for potential innovation opportunities, voluntarily make more constructive contributions to the organization, set more challenging tasks and goals based on the interests of the organization, and have more courage and confidence to realize their work pursuit with the support of dual leadership (Tumasjan and Braun, 2012). Furthermore, dual leadership can effectively stimulate employees’ promoting regulatory focus. Promoting regulatory focus employees are committed to adopting novel methods for creative behavior. Compared with defensive regulatory focus, they pay more attention to the achievement of achieving goals rather than the cost of avoiding losses. Under the influence of promoting regulatory focus, employees have more confidence in predicting and achieving challenging goals and ability, individual behavior controllability assessment and behavior cost perception will stimulate employees’ strong motivation to implement constructive behavior (Parker et al., 2010), even in the case of external environment obstacles, because dual leadership evokes employees’ positive self-concept cognition, employees with promoting regulatory focus will strive to overcome difficulties and adhere to constructive deviant behavior under the hint of positive self-concept. Therefore, employees with higher promoting regulatory focus have higher ability and cognitive motivation and dare to break outdated organizational rules, take promoting the interests of the organization as its own responsibility, and finally actively implement constructive deviant behaviors. Based on this, the following assumptions are put forward:

H2: Promoting regulatory focus plays an intermediary role between dual leadership and constructive deviant behavior.

### Mediating Role of Role Width and Self-Efficacy

Leadership is an important external variable to stimulate employees’ behavior motivation. Open leadership encourages employees to exchange and share knowledge, which aims to stimulate employees’ critical thinking, support employees to break through the fixed mode, make employees feel the innovation support of the organization, and enhance employees’ psychological identity with the organization (Mom et al., 2010), employees are allowed to put forward and practice risky ideas, increase subordinates’ inspiration for diversified approaches and ideas, and tend to stimulate employees’ pursuit of behavior variation and experience innovation, so as to promote employees’ organizational exploration behavior (Gupta et al., 2006). Open leadership – closed leadership effectively controls employees’ work direction and process according to rules and plans, avoids contradictions and conflicts among members, coordinates employees’ behaviors in an orderly manner, sets clear mission objectives for organizational interests, pays attention to the predictability and understandability of employees’ behaviors, and deepens employees’ understanding of the innovative direction and market (Mom et al., 2010), further stimulate employees’ pursuit of stable experience and innovative results, so as to promote employees’ utilization behavior (Gupta et al., 2006). The daily interaction between leaders and employees is an important benchmark for employees to know themselves. Dual leadership management provides employees with another layer of institutional guarantee in addition to their perceived freedom of behavior and gives employees a sense of freedom.

In work, constructive behavior is often the activity behavior outside the individual’s own work. Dual leadership makes employees feel that engaging in affairs outside their roles can obtain a sense of job achievement and be appreciated by leaders. When employees are supported and encouraged to carry out constructive and positive organizational behavior, and the behavior results are fed back and recognized by leaders. Employees will maintain a consistent position with the interests of the organization and commit themselves to actively making more constructive contributions to the organization, so as to generate a perception that they can be competent or complete other affairs outside the specified scope of work, and stimulate employees’ high level of role width and self-efficacy. Existing studies on role breadth self-efficacy mainly include individual and situational aspects, such as self-esteem at the individual level (Parker and Sharon, 1998), active personality (Parker et al., 2006), leadership style at the situational level, organizational climate and work characteristics (Sonnentag and Spychala, 2012). However, the mechanism and boundary effect of dual leadership at the individual level on employees’ psychological motivation have not been systematically explained. According to the social cognitive theory, self-efficacy is conducive to encouraging employees to give full play to their individual initiative and initiative, and is conducive to employees to carry out constructive and positive organizational behavior (Bandura, 1978). Role width self-efficacy enhances employees’ predictability and controllability of behavior results, so they also have stronger expectations and desires for goals. The internal motivation of psychological cognition has become an important driving force for employees to take behavior. Following this logic, high role width self-efficacy gives employees confidence and motivation to play a variety of work roles, which means that individuals voluntarily assume a wider range of responsibilities and risks. Employees’ enthusiasm and probability of putting forward innovative ideas to improve the current situation of the organization have increased, laying a foundation for employees to adopt constructive deviant behaviors. When employees are committed to making constructive contributions to the organization, even if they encounter obstacles and difficulties, they will firmly believe that they will continue to carry out constructive behavior even if they take deviant ways with the support of dual leaders. To sum up, dual leadership, as an environmental factor, affects employees’ behavior by affecting employees’ psychological cognitive state (Zhang et al., 2016). Role width self-efficacy is a psychological cognitive state of employees, and dual leaders provide certain planned action support and guarantee for employees. Following this logic, they create a supportive psychological environment in the organization, which is conducive to shaping employees’ cognition of their own abilities, especially improving employees’ role width self-efficacy. After employees have role width self-efficacy, they will actively identify the problems and opportunities existing in the organization and take the initiative to make constructive out of role behaviors for the organization (Parker and Sharon, 1998). When employees are committed to making constructive contributions to the organization, even if they encounter obstacles and difficulties, they will firmly believe that they will continue to carry out constructive behavior even if they take deviant ways with the support of dual leaders. To sum up, dual leadership, as an environmental factor, affects employees’ behavior by affecting employees’ psychological cognitive state (Zhang et al., 2016). Role width self-efficacy is a psychological cognitive state of employees, and dual leadership provides employees with certain planned action support and guarantee, and creates a supportive psychological environment in the organization, which is conducive to shaping employees’ cognition of their own abilities, especially improving employees’ role width self-efficacy. Therefore, dual leadership can affect employees’ constructive deviant behavior through employees’ role width and self-efficacy. Therefore, this study puts forward hypotheses.

H3: Role width self-efficacy plays an intermediary role between dual leadership and constructive deviant behavior.

### Chain Mediating Role of Promoting Regulatory Focus and Role Width Self-Efficacy

Existing studies have confirmed that psychological factors often do not play a single isolated role in the relationship between leaders and members, but multiple intermediary roles. The interaction between psychological factors is extremely complex, and the intermediary chain model can provide a more dynamic and complete action path mechanism. Regulatory focus is induced by information cues in the environment and task framework and acts as an intermediary variable in many action mechanisms. Leadership behavior can affect employees’ attitudes and behaviors through their adjustment focus. As the key factor of work self-regulation in the environmental factors of dual leadership (Lanaj et al., 2012), facilitative regulatory focus is employees’ positive psychological cognition, which affects individuals’ sense of control over target tasks, and the sense of control is significantly related to the sense of efficacy (Judge et al., 2002). Specifically, employees with facilitative regulatory focus tend to actively find and collect problem information, try various solutions to solve problems and improve the current situation of the organization (Friedman et al., 2001), have a high risk preference, be not afraid of challenges, and have the courage to assume the responsibility of implementing behaviors outside the role. Under the positive psychological cognition of promoting regulatory focus, employees voluntarily assume more responsibilities outside their own work and tend to play more work roles to give play to the subjective initiative of constructive behavior. Employees have a greater sense of responsibility and mission. They are willing to make more constructive contributions to the organization and hope to achieve mutual benefit in the exchange process (Podsakoff et al., 1996). When employees establish good interaction with leaders, and have strong expectations and hopes for the future blueprint of the organization, they are committed to pursuing the sense of achievement of creative results of work, and the satisfaction of psychological needs can stimulate employees’ internal motivation and psychological security. Motivation and security enable employees to form a high level of role width self-efficacy. High role width self-efficacy, it can improve the predictability and control of employees’ behavior success, and employees have stronger motivation to take constructive action. Driven by positive psychological internal motivation, employees have more confidence to face challenges and risks, and can stimulate positive desire to try, so as to give full play to individual initiative and take the initiative to carry out constructive deviant behavior. Based on this, this article puts forward the following assumptions:

H4: Promoting regulatory focus, role width and self-efficacy play a chain intermediary role between dual leadership and constructive deviant behavior.

**FIGURE 1:**
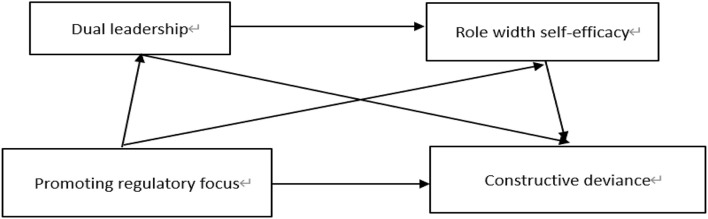
Theoretical model between dual leadership and constructive deviant behavior.

To sum up, the theoretical model is shown in Figure 1.

## Research Design

### Research Samples and Data Collection

This study recruits participants through the university alumni network. This sampling method can invite employees and managers from different industries and enterprises to participate in the survey, which helps to increase the external validity of the research conclusions (Qin et al., 2017). Specifically, a total of 350 enterprise workers were recruited through the online questionnaire. We first introduced the purpose of the survey to the leaders of each work team, and emphasized the confidentiality of the survey and the importance of truthful answers. Then each leader provided the list and e-mail address of all members of the team, and obtained the names of 350 workers through the above methods (Podsakoff et al., 2012). Then, the researchers sent the electronic link of the questionnaire to the e-mail of each employee and leader, respectively. This survey method covering all members of the team can avoid potential sampling deviation. The purpose of the survey is introduced again in the guidance of each e-questionnaire, and the confidentiality of the survey and the importance of real answer are emphasized. When filling in the questionnaire, the staff need to report their own information demographic characteristics, recognition perception of dual leadership, role width self-efficacy perception, promoting regulatory focus and constructive deviant behavior. After the questionnaire was collected, we sorted out the questionnaire according to the e-mail address filled in the questionnaire, eliminated the invalid questionnaires with too short filling time and obvious response tendency, and finally obtained 333 valid employee questionnaires, which were valid the rate reached 95.14%.

In the final valid employee sample, participants come from multiple work organizations. Among them, there are 56 people from state-owned enterprises, accounting for 16.82%, 215 people from private enterprises, accounting for 64.56%, 37 people working in foreign-funded enterprises, accounting for 11.11%, and finally 25 people affiliated to government organs, accounting for 7.50%, of which women account for 49.25% and men account for 50.75%. Most workers are between 25 and 30 years old, accounting for 60.06%. Table 1 describes the basic demographic characteristics and basic information of the study sample.

**TABLE 1:** Sample demographic characteristics (*n* = 333).

**Statistical variables**	**Classification**	**Frequency**	**Percentage**
1. Gender	Male	169	50.75%
	Female	164	49.25%
2. Age	Under 25	26	7.81%
	25–30 years old	200	60.06%
	31–40 years old	84	25.23%
	36–40 years old	23	6.90%
3. Education level	Junior college	42	12.61%
	Undergraduate	264	79.28%
	Master	26	7.81%
	Doctor	1	0.30%
4. Working years	1∼3 years	55	16.52%
	3∼5 years	91	27.33%
	More than 5 years	180	54.05%
5. Nature of employee unit	State-owned enterprise	56	16.82%
	Private enterprise	215	64.56%
	Foreign enterprise	37	11.11%
6. Employee position category	Government-affiliated institutions	25	7.51%
	Ordinary staff	128	38.44%
	Grass roots management	130	39.04%
	Middle manager	67	20.12%
	Senior management	8	2.40%

It can be seen from Table 1 that among the surveyed employees, men account for 50.75%, women account for 49.25%, and the proportion of men and women is the same. In terms of age distribution, the sample employees are basically under the age of 40, which is in line with the age characteristics of the new generation of employees. In this study, 87.39% of the employees with higher educational background were bachelor degree or above, that is, most of them were well-educated. The nature of employees is that state-owned and state holding enterprises account for 16.82%, private enterprises account for 64.56%, foreign-funded enterprises account for 11.11%, and public institutions account for 7.51%. The staff positions are mainly ordinary staff and grass-roots management staff, accounting for 38.44 and 39.04%, respectively, middle-level managers accounting for 20.12% and senior managers accounting for 2.40%. The overall statistical results basically reflect the current situation of the management personnel and the new generation of employees of the enterprise.

### Variable Measurement

The measurement scales of all constructs are from the mature scales in the research literature of international important journals, and adopt the standard translation back translation procedure (Brislin, 1980). The instruments to be measured in this study include dual leadership, role width, self-efficacy, regulatory focus, and constructive deviant behavior. Likert seven point scoring method was adopted for all scales, and from 1 to 7, it means “very disagree” to “very agree.”

(1) Open leadership: The measurement of dual leadership is based on the open leadership (OL) and closed leadership (CL) scale compiled by Rosing et al. (2011), which contains 14 items. Seven of these items measure open leadership, such as “my leadership encourages employees to experiment with new ideas,” Cronbach’s α the coefficient is 0.886. The other seven items measure closed leadership, such as “my leader will establish some guidelines and norms in the work process,” Cronbach’s α the coefficient is 0.819.
(2) Facilitative regulatory focus: Adopt the two-dimensional scale of Neubert et al. (2008) and measure the facilitative regulatory focus with nine items, such as “if I have the opportunity to participate in projects with high risk and high return, I will certainly accept it,” Cronbach’s α the coefficient is 0.898.
(3) Role width self-efficacy: Parker’s (Parker and Sharon, 1998) seven item questionnaire was used to measure role width self-efficacy. Representative items include “designing new work procedures in your own work field,” Cronbach’s α the coefficient is 0.820.
(4) Employees’ constructive deviant behavior: Adopt the Galperin and Bella (2012) scale with nine items, such as “in order to promote organizational development, he/she will point out colleagues’ mistakes in work,” Cronbach’s α the coefficient is 0.822.
(5) Control variables: Gender, age, educational background, years of service, nature of unit, and position were included in the control variables.

## Data Analysis and Results

### Distinguish Validity Test and Common Method Deviation Test

This study conducted confirmatory factor analysis through Amos22.0 to test the discriminant validity of open leadership, closed leadership, promoting regulatory focus, role width, self-efficacy and constructive deviant behavior. As shown in Table 2, each fitting index of the five factor model meets the critical standard, and the fitting effect of the model is significantly better than that of other models. The analysis results show that the discrimination validity among the five variables is high, and the fitting effect of the five factor model is good.

**TABLE 2:** Discriminant validity analysis (*n* = 333).

**Model**	**χ ^2^**	**df**	**χ ^2^/df**	**NFI**	**RMSEA**	**CFI**
Five factor model	1198.194	725	1.693	0.814	0.044	0.916
Four factor model	1795.585	730	2.460	0.721	0.066	0.812
Three factor model	2190.113	734	2.984	0.660	0.077	0.743
Two factor model	2966.125	816	3.635	0.566	0.089	0.640
Single factor model	3886.501	859	4.524	0.449	0.103	0.508

*Five factor sub model: Open leadership, closed leadership, promoting regulatory focus, role width, self-efficacy, constructive deviant behavior.*

*Four factor model: Open leadership + closed leadership, promoting regulatory focus, role width, self-efficacy, constructive deviant behavior.*

*Three factor model: Open leadership + closed leadership + promoting regulatory focus, role width, self-efficacy and constructive deviant behavior.*

*Two factor model: Open leadership + closed leadership + promoting regulatory focus + role width, self-efficacy and constructive deviant behavior.*

*Single factor model: Open leadership + closed leadership + promoting regulatory focus + role width self-efficacy + constructive deviant behavior + represents the synthesis as a factor.*

As for the common method deviation, on the one hand, anonymous questionnaire and other methods are used to control in advance. On the other hand, the Harman one-way test was used to test. The results showed that the cumulative variance contribution rate of the first factor was 22.376% <40%, indicating that there was no homologous deviation in the study.

### Correlation Analysis and Inspection

The results of correlation analysis showed the correlation coefficient, mean and variance of each variable. As shown in Table 3, in which dual leadership had a significant positive correlation with promoting regulatory focus (*r* = 0.394, *P* < 0.01), role width self-efficacy (*r* = 0.462, *P* < 0.01) and constructive deviant behavior (*r* = 0.284, *P* < 0.01). At the same time, promoting regulatory focus had significant positive effects on role width self-efficacy (*r* = 0.498, *P* < 0.01) and constructive deviant behavior (*r* = 0.373, *P* < 0.01). Finally, there was a significant positive correlation between role width self-efficacy and constructive deviant behavior (*r* = 0.365, *P* < 0.01).

**TABLE 3:** Correlation analysis results of research variables (*n* = 333).

**Variable**	**1**	**2**	**3**	**4**	**5**	**6**	**7**	**8**	**9**	**10**
1. Your gender	1									
2. Your age	0.01	1								
3. Your education level	0.05	0.09	1							
4. Your working hours	–0.03	0.579**	0.03	1						
5. Nature of your organization	0.07	–0.03	0.08	–0.10	1					
6. Position level of your organization	−0.264**	0.233**	0.184**	0.255**	–0.10	1				
7. Dual leadership	–0.04	0.02	0.07	0.04	0.144**	0.197**	1			
8. Role width self-efficacy	−0.131*	0.125*	0.131*	0.126*	0.07	0.404**	0.462**	1		
9. Promoting focus	−0.111*	–0.00	0.04	0.02	0.05	0.170**	0.394**	0.498**	1	
10. Constructive deviance	–0.07	–0.03	0.07	–0.06	0.02	0.11	0.284**	0.373**	0.365**	1
Mean value	1.49	2.31	1.96	3.33	2.09	1.86	4.09	4.89	5.01	4.37
Variance	0.501	0.715	0.464	0.825	0.757	0.816	1.068	1.015	1.008	0.961

*P < 0.05. **P < 0.01 (double tailed), and the result in diagonal brackets is Cronbach’s α.

### Hypothesis Test

#### Main Effect Test

In order to test the impact of dual leadership on the constructive deviant behavior of the new generation of employees, the dual leadership defined as dependent variable and independent variable were analyzed by regression. According to the results of model 1 and model 2 in Table 4, dual leadership has a significant positive impact on employees’ constructive deviant behavior (β = 0.277, *P* < 0.001).

**TABLE 4:** Intermediary test of adjustment focus.

**Variable**	**Promoting regulatory focus**		**Constructive deviance**		
	**M1**	**M2**	**M3**	**M4**	**M5**
Gender	–0.073	–0.073	–0.048	–0.049	–0.027
Age	–0.039	–0.025	–0.0055	0.005	0.0125
Education level	0.015	0.007	0.053	0.048	0.046
Working hours	0.001	–0.007	–0.090	–0.096	–0.094
Unit nature	0.069	0.008	0.019	–0.027	–0.029
Post-level	0.163	0.085	0.109	0.050	0.025
Dual leadership		0.373***		0.277***	0.168
Promoting regulatory focus					0.293***
*R* ^2^	0.040	0.170	0.025	0.096	0.167
*F*	2.275**	9.509***	1.377	4.940***	8.137***

*P < 0.05, **P < 0.01, ***P < 0.001 (two tails).

#### Facilitating Adjustment Focus Intermediary Inspection

This study uses hierarchical regression to test the mediating role of constructive responsibility perception. The relevant results are shown in the table. Firstly, it can be seen from model 1 and model 2 that dual leadership has a positive impact on promoting regulatory focus (β = 0.373, *P* < 0.001). Secondly, it can be seen from model 3 and model 4 that dual leadership has a significant positive effect on constructive deviant behavior (β = 0.277, *P* < 0.001). In addition, taking dual leadership and promoting regulatory focus as independent variables, we do regression analysis on employees’ constructive deviant behavior. It can be seen from model 4 and model 5 that dual leadership (β = 0.168, *P* > 0.05) had no significant positive effect on employees’ constructive deviant behavior, and promoted regulatory focus (β = 0.293, *P* < 0.001). It shows that the promoting regulatory focus plays a complete intermediary role between dual leadership and employees’ constructive deviant behavior, and the hypothesis has been verified.

#### Role Width Self-Efficacy Mediation Test

In this study, hierarchical regression was used to test the mediating role of role width self-efficacy. The relevant results are shown in the Table 5. Firstly, according to model 6 and Model 7, dual leadership has a positive impact on role width and self-efficacy (β = 0.389, *P* < 0.001). Secondly, according to model 8 and model 9, dual leadership has a significant positive effect on constructive deviant behavior (β = 0.277, *P* < 0.001). In addition, taking dual leadership and role width self-efficacy as independent variables, this article makes a regression analysis on employees’ constructive deviant behavior. It can be seen from model 9 and model 10 that knowledge employees are embedded in work (β = 0.147, *P* > 0.05) had no significant positive effect on employees’ deviant innovation behavior, role width and self-efficacy (β = 0.333, *P* < 0.001) had a significant positive impact on employees’ deviant innovation behavior. It shows that role width self-efficacy plays a complete intermediary role between knowledge workers’ job embeddedness and employees’ deviant innovation behavior, and the hypothesis is verified.

**TABLE 5:** Intermediary test of role width self-efficacy.

**Variable**	**Role width self-efficacy**		**Constructive deviance**		
	**M6**	**M7**	**M8**	**M9**	**M10**
Gender	–0.040	–0.040	–0.048	–0.049	–0.035
Age	0.020	0.034	–0.005	0.005	–0.006
Education level	0.050	0.043	0.053	0.048	0.033
Working hours	0.025	0.015	–0.090	–0.096	–0.101
Unit nature	0.111	0.047	0.019	–0.027	–0.043
Post-level	0.384***	0.302***	0.109	0.050	–0.050
Dual leadership		0.389***		0.277***	0.147
Role width self-efficacy					0.333***
*R* ^2^	0.181	0.322	0.025	0.096	0.171
*F*	11.985***	22.066***	1.377	4.94***	8.37***

* *P* < 0.05, **P < 0.01, ***P < 0.001.

#### Chain Intermediary Inspection

In this study, the bootstrap method is used to verify the chain mediation. The specific results are shown in Table 6. According to the upper and lower limits of the confidence interval in the table, the indirect impact of dual leadership on employees’ constructive deviant behavior is significant (the effect value is 0.0253). The effect estimates of each path are within the confidence interval, and the confidence interval does not contain 0, indicating that the mediating effects of different paths are significant. Among them, the effect value of promoting regulatory focus and role width self-efficacy as chain mediators is 0.0050 (0.0003, 0.0117), and the range does not include 0. Therefore, promoting regulatory focus and role width self-efficacy play a significant chain mediating role in the relationship between dual leadership and constructive deviant behavior.

**TABLE 6:** Chain intermediary test.

**Effect path**	**Effect value**	**Standard error**	**95% confidence interval**	
			**Lower limit**	**Upper limit**
Indirect effect of X on Y	0.0253	0.0046	0.0166	0.0347
X→M_1_→Y	0.0116	0.0034	0.0052	0.0186
X→M_2_→Y	0.0093	0.0032	0.0039	0.0165
X→M_1_→M_2_→Y	0.0050	0.0029	0.0003	0.0117

*X refers to dual leadership; Y is constructive deviant behavior; M1 is the promoting adjustment focus; M2 is role width self-efficacy.*

## Conclusion and Enlightenment

### Research Conclusion

With the advent of the era of knowledge economy, different enterprises have different management modes for the new generation of employees, which will lead to different psychological cognition and organizational behavior reactions of employees. Based on this, based on information evaluation theory and social cognitive theory, this study explores the theoretical mechanism of dual leadership affecting constructive deviant behavior, focuses on the intermediary effect of promoting regulatory focus and role width self-efficacy, and obtains some valuable conclusions: dual leadership is an important factor positively affecting employees’ constructive deviant behavior. The role path is to stimulate employees’ positive psychological cognition, promote employees’ constructive behavior motivation, promote type adjustment focus and role width, and play a complete intermediary role in self-efficacy. Moreover, the intermediary chain composed of employees’ promoting regulatory focus and role width self-efficacy plays a significant chain intermediary role in the impact of dual leadership on employees’ constructive deviant behavior.

### Theoretical Significance

(1) The results show that dual leadership has a positive impact on employees’ constructive deviant behavior. Paradox cognition promotes dual leadership to seek the balance point of Inter Organizational development. The dynamics of leadership behavior is consistent with the changes of external situations. The complementary leadership model is deeply combined with mutually coupled behavior strategies to stimulate employees’ positive organizational behavior on the basis of rigid and flexible leadership. Although some scholars have preliminarily verified that dual leadership can stimulate employees’ positive organizational behavior and improve organizational effectiveness (Rosing et al., 2011), it mainly focuses on the team and organization research at the macro level, and there is still a lack of individual level research on situational embedding. This study creatively combines the role width self-efficacy under the internal motivation mechanism of dual leadership affecting the effect with the promoting regulatory focus under the self-regulation mechanism, deepens the macro strategic research on organizational duality to the micro organizational behavior research field, and confirms that dual leadership is an important predictor of the constructive deviant behavior of the new generation of employees. It reveals the internal mechanism of dual leadership affecting employees’ constructive deviant behavior, and provides a new research paradigm for subsequent related variables.

(2) This study introduces variable promoting regulatory focus and role width self-efficacy, which enriches the research on the influence path of dual leadership on employees’ constructive deviant behavior. It is known that dual leadership collaborative management of employee behavior can make employees seek a balance between rashness and stability, and make employees’ constructive deviant behavior not only have the innovative significance of active change, but also have the rigorous spirit of seeking stability in risk. With the help of dual thinking foundation, integrate the internal logical relationship between situation cognition behavior, according to social cognition theory. Under the influence of dual leadership, the new generation of employees stimulate positive psychological motivation, and psychological motivation, as an important factor affecting employees’ behavior, promotes employees to carry out meaningful and constructive deviant behavior. The research results reveal the internal psychological “black box” mechanism of dual leadership affecting constructive deviant behavior, provide certain theoretical value and guiding significance for the innovative management of the new generation of employees, and theoretically enrich the leadership mechanism, especially the research on the influence mechanism of subordinate active organizational behavior.

(3) The influence of dual leadership on the constructive deviant behavior of the new generation of employees can not be regarded as a single action mechanism in isolation. The interaction between different psychological cognition of employees in the organization significantly affects the motivation of constructive deviant behavior. Promoting regulatory focus can effectively stimulate employees’ role width self-efficacy, which further stimulates the willingness of the new generation of employees to take constructive deviant behavior. By exploring the chain multiple mediating role of promoting regulatory focus and role width self-efficacy, it provides a more dynamic and complete path mechanism for dual leadership to affect employees’ constructive deviant behavior. Therefore, this article introduces the promoting regulatory focus and role width self-efficacy into the internal mechanism of employees’ deviant innovation behavior in the form of intermediary chain, which provides a new reference perspective for subsequent related research and has certain innovative significance.

### Practical Enlightenment

Firstly, this study explores the mechanism of dual leadership on constructive deviant behavior, and proves that dual leadership can promote constructive deviant behavior through positive psychological cognition. Therefore, enterprises are encouraged to set up a scientific and reasonable dual leadership model to guide the new generation of employees to embark on a formal and standardized path of constructive deviant behavior under the control of positive psychological motivation. Open leadership encourages employees to jump out of the comfort circle, break the original thinking and think about things from a new perspective. Closed leadership establishes rules and regulations, and instructors focus on the completion of tasks. The sense of identity and security provided by dual leaders to employees is conducive to employees to use resources to complete innovative tasks. Therefore, dual leaders need to support employees to put forward constructive opinions, give employees certain initiative and decision-making power, and provide appropriate work autonomy space, so as to facilitate employees to give full play to their work value, overcome the paradox of organizational innovation and achieve subversive creative results.

Secondly, dual leadership can stimulate employees’ positive internal psychological cognition, help to cultivate employees’ sense of “ownership,” promote employees to form self-identity and cultivate emotional connection with the organization. Because the new generation of employees have distinct personality characteristics, leaders need to combine the macro strategic concept with the micro organizational behavior characteristics of the new generation of employees. The strategic formulation and practical measures should conform to the characteristics of employees’ pursuit of innovation and courage to explore, and take specific management measures according to the unique cultural values of the new generation of employees. For example, formulate personalized career planning for employees, encourage employees to carry out self-career management, provide creative resources and standardized institutional guarantee for employees, customize internal incentive remuneration to meet the needs of internal sense of achievement, and adopt flexible working system and group rotation system suitable for employees’ personality in time and space, give full play to the subjective initiative of the new generation of employees and encourage employees to actively change and innovate.

Finally, employees’ positive psychological cognition stimulates positive constructive deviant behavior. The supportive resources and flexible encouragement provided by dual leaders promote employees’ promoting regulatory focus cognition and self-efficacy perception, and provide rich support and guarantee to help employees manifest their internal motivation. Therefore, enterprises need to encourage employees to take reasonable and constructive measures against deviant behavior, such as dual leadership using the organization’s work resources (such as promotion benefits, internal, and external incentives, etc.) and non-work resources (such as organizational support, identity atmosphere, etc.) to help employees better carry out creative work and avoid the negative phenomenon of job burnout caused by the opposition of superiors. The organization needs to create a good cultural atmosphere of tolerance, match the personalized characteristics of the new generation of employees who are not afraid of risks and dare to challenge, encourage employees to realize self-worth, help enterprises break through bottlenecks, achieve change and upgrading, make common progress with enterprises, achieve goals and achieve win-win results. Pay attention to the new generation of employees’ pursuit of fairness in organizational distribution, management process and interpersonal communication, avoid oppressive leadership and other traditional methods, adopt flexible and scientific dual leadership mode, create a good environment for the new generation of employees to give full play to their abilities, and strive to stimulate employees’ self-efficacy perception. In addition, training should be conducted for the new generation of employees to strengthen their psychological capital such as brevity when they encounter difficulties, so as to provide favorable conditions for the new generation of employees to play a stable and healthy role in the organization. Managers should actively help the new generation of employees correctly understand themselves and improve their work and life experience. Through positive feedback, the new generation of employees can maintain a high perception of self-efficacy, maintain their recognition of organizational goals, and have a sense of responsibility for their work. This study proves that the self-efficacy of the new generation of employees plays a fundamental role in these aspects. Therefore, when selecting and training the new generation of employees, the human resources management departments of enterprises and other organizations should start with personality traits and psychological cognition, and focus on selecting individuals with more positive self-efficacy. For the existing new generation of employees, they should not give up the shaping of positive psychological cognition and values, and should shape a positive working attitude for the new generation of employees through organizational socialization and other means, enhance the self-efficacy and work enthusiasm of the new generation of employees at work.

### Research Deficiency and Prospect

Based on social information parity theory and social cognitive theory, this article explores the relationship between dual leadership and employees’ constructive deviant behavior, considers the intermediary role of promoting regulatory focus and role width self-efficacy, and obtains some valuable conclusions, but there are still some deficiencies.

Firstly, this study only examines the impact effect and action process of dual leadership on employee behavior. The research perspective mainly focuses on the individual level. In the future, we can explore the overall effect and action mechanism of dual leadership on leaders themselves, teams and organizations, and consider the more comprehensive and systematic impact effect of dual leadership under the influence of external factors of organizational situation.

Secondly, future research can further fit the Chinese situation, consider further supplementing and improving the research model and adding research variables in line with China’s national conditions, such as the golden mean, confucian culture and other factors with Chinese management characteristics into the study of employees’ organizational behavior, so as to provide theoretical guidance for the management practice of domestic new generation employees according to local conditions. The purpose is to further verify the effectiveness of dual leadership in China’s enterprise management practice.

## Data Availability Statement

The original contributions presented in the study are included in the article/supplementary material, further inquiries can be directed to the corresponding author.

## Ethics Statement

The studies involving human participants were reviewed and approved by the Ethics Committee of Beijing Technology and Business University. The patients/participants provided their written informed consent to participate in this study.

## Author Contributions

YZ selected the theme, planned the framework, and grasped the direction. HQ collected the data, wrote the manuscript, and analyzed the literature. Both authors contributed to the article and approved the submitted version.

## Conflict of Interest

The authors declare that the research was conducted in the absence of any commercial or financial relationships that could be construed as a potential conflict of interest.

## Publisher’s Note

All claims expressed in this article are solely those of the authors and do not necessarily represent those of their affiliated organizations, or those of the publisher, the editors and the reviewers. Any product that may be evaluated in this article, or claim that may be made by its manufacturer, is not guaranteed or endorsed by the publisher.
